# Burnout Syndrome Among Primary Care Physicians in Romania: A National Overview

**DOI:** 10.3390/healthcare13222966

**Published:** 2025-11-19

**Authors:** Nina Ciuciuc, Eugenia-Maria Lupan-Mureșan, Alexandra-Ioana Roșioară, Monica Popa, Dana Manuela Sîrbu, Daniela Curșeu, Codruța Alina Popescu, Bogdana-Adriana Năsui

**Affiliations:** 1 Department of Community Medicine, “Iuliu Hațieganu” University of Medicine and Pharmacy, 400349 Cluj-Napoca, Romania; nina.ciuciuc@umfcluj.ro (N.C.); alexandra.rosioara@umfcluj.ro (A.-I.R.); monica.popa@umfcluj.ro (M.P.); dsirbu@umfcluj.ro (D.M.S.); dcurseu@umfcluj.ro (D.C.); adriana.nasui@umfcluj.ro (B.-A.N.); 2 Research Center in Preventive Medicine, Health Promotion and Sustainable Development, “Iuliu Hațieganu” University of Medicine and Pharmacy, 400349 Cluj-Napoca, Romania; 3 Department 6—Surgery, Emergency Medicine Discipline, “Iuliu Hațieganu” University of Medicine and Pharmacy, 400006 Cluj-Napoca, Romania; 4 Emergency Department, County Emergency Hospital Cluj-Napoca, 400006 Cluj-Napoca, Romania; 5 Department of Practical Abilities—Human Sciences, “Iuliu Hațieganu” University of Medicine and Pharmacy, 6 Louis Pasteur Street, 400349 Cluj-Napoca, Romania; cpopescu@umfcluj.ro

**Keywords:** burnout, emotional exhaustion, family medicine, emergency medicine, Romania, predictors, psychological stress

## Abstract

**Background/Objectives:** This study provides the first national-level assessment of burnout among family physicians (FPs) and emergency physicians (EPs) in Romania. Burnout syndrome is a growing concern in medical settings, particularly among primary care physicians. The aim of this study was to compare the levels of frustration and emotional exhaustion (EE) among the Romanian EPs and FPs. **Methods:** A cross-sectional observational online study was conducted using validated psychometric questionnaires. Statistical analyses included correlation, regression, and between-group comparisons. **Results:** A total of 307 participants were included (222 FP and 85 EP). EPs reported significantly higher levels of frustration (Mean ± SD = 1.94 ± 1.22) and EE (2.52 ± 0.86) than FPs (2.01 ± 1.24 and 2.51 ± 0.84, respectively; *p* < 0.001). Regression analysis identified ‘feeling at the end of one’s strength’ (M = 1.26, *p* < 0.001) and ‘evening exhaustion’ (M = 1.20, *p* = 0.0112) as significant predictors of EE. **Conclusions:** The findings highlight the need for differentiated intervention strategies adapted to the specific work conditions of each medical specialty. Organizational support and mental health promotion are recommended to reduce burnout, alongside encouraging professionals to practice individual strategies to ensure well-being.

## 1. Introduction

Burnout amongst healthcare professionals (HCPs) is a global phenomenon that poses a significant impact on the quality of medical care, patient safety, and provider well-being [1,2]. Conceptualized by Freudenberger and refined by Maslach and Jackson [3], burnout syndrome is defined by emotional exhaustion (EE), depersonalization (DP) and reduced personal accomplishment (PA). Studies have shown that physicians display higher levels of burnout in comparison with other professions [4], a tendency exacerbated by the recent COVID-19 pandemic [5,6].

Across Europe, burnout prevalence among physicians ranges between 25% and 60%, depending on medical specialty and measurement instruments [7,8,9]. In Romania, studies are limited on this topic, yet existing data suggest a worrisome tendency. A report by the Romanian College of Physicians indicated that approximately 70% of physicians have experienced symptoms of professional exhaustion [10], whilst more recent studies have indicated high burnout rates among emergency physicians (EPs), ranging between 43% and 54% [11,12,13].

Factors leading to burnout include excessive workloads, lack of control over one’s work, conflicts with patients, work–personal life imbalance, alongside individual traits such as perfectionism or lack of effective coping strategies [14,15]. Particularly, strenuous human interactions and frustration regarding one’s professional role were identified as relevant predictors of EE [16]. In this context, “frustration” refers to the perceived mismatch between professional goals and the systemic or organizational constraints that hinder their fulfillment, a feeling often associated with reduced personal accomplishment. Protective factors, such as a sense of professional efficiency, social support and physical activity, can contribute to burnout prevention [17,18].

Most of the international research focuses on hospital-based secondary care physicians, including residents. As such, burnout in the primary care sector is understudied, both for family physicians (FPs) and EPs [19]. The dissimilarities of these two specialties—in terms of workload and the frequency of overcrowding, the pattern of direct patient care, administrative duties and the unconcealed facing of acute suffering—justify the need for a personalized analysis of the impact of burnout and frustration on these professionals.

Physician burnout has been widely documented in Western Europe, where it has been linked to workload intensity, workforce aging, and gender disparities. However, evidence from Central and Eastern Europe remains limited, leaving a gap in the understanding of the phenomenon in health systems facing similar or greater structural pressures. One recent study from Ukraine addressed burnout among EPs during the COVID-19 pandemic, highlighting gender differences in burnout risk [20]. Nonetheless, such investigations remain scarce compared to Western European data. By focusing on Romania, this study is aimed at addressing that gap and positioning the findings within the wider European public health landscape.

At the European level, the Mental Health of Nurses and Doctors Survey (MeND), conducted by the World Health Organization in 2024–2025, provided up-to-date data on the mental health of physicians and nurses in the 27 EU Member States, Iceland, and Norway. The report highlights a high prevalence of mental health problems and unsafe working conditions among healthcare personnel, including extended working hours, workplace violence, and lack of institutional support, all of which are associated with poorer mental health outcomes. These findings emphasize the importance of situating national studies within the broader European context and justify the need to explore these issues among Romanian physicians [21].

This study aims to analyze the levels of EE and frustration among Romanian FPs and EPs, as well as their correlations with certain factors: individual (age, gender, lifestyle), setting (work environment), and occupational (workload, interpersonal stress). Through this research, we hope to contribute to a better understanding of the determinants of burnout for the abovementioned professional groups, whilst outlining possible directions for organization interventions and tailored health policies.

## 2. Materials and Methods

The current study is an observational, cross-sectional investigation conducted using an adapted Romanian version of the Maslach Burnout Inventory (MBI). This version is based on the structure of the MBI-HSS (Medical Personnel) [3] and evaluates the three core dimensions of burnout: emotional exhaustion, depersonalization (or cynicism), and reduced personal accomplishment (sometimes described in later adaptations as reduced professional efficacy).

To tailor the instrument to the objectives of this study, additional items regarding eating behaviors were incorporated, resulting in an adapted version used for academic research purposes.

The Romanian version of the Maslach Burnout Inventory was linguistically and culturally adapted and pretested on a sample of 30 participants to ensure clarity and relevance in the local medical context. The three core constructs—EE, DP, and reduced PA—are conceptually well aligned with their original definitions and are widely recognized among Romanian HCPs. Although a full transcultural validation was beyond the scope of this study, pretesting helped ensure that the instrument was culturally appropriate for this population.

The pretest sample consisted of 30 physicians and medical residents from the same target population, selected through convenience sampling. Participants completed the survey in the same format and under the same conditions as the main study, allowing us to assess clarity, feasibility, and internal consistency. Informal feedback on language and item structure was also collected, supporting the cultural and linguistic appropriateness of the adapted version. The Spearman correlation coefficient was used to evaluate viability, with a value of ρ = 0.82, indicative of a good correlation between variables. The Cronbach α coefficient was used to determine the internal consistency of the questionnaire, with its value of 0.88 indicating a good internal consistency.

The survey was written and administered in Romanian, the native language of all the participants, and was disseminated online through the professional network, with the support of the Romanian College of Physicians. The time needed for the completion of the survey was 25–30 min. All study participants provided informed consent at the time of survey completion. Data were collected through a secure online platform, Google Forms, between December 2024 and March 2025.

**Figure 1:**
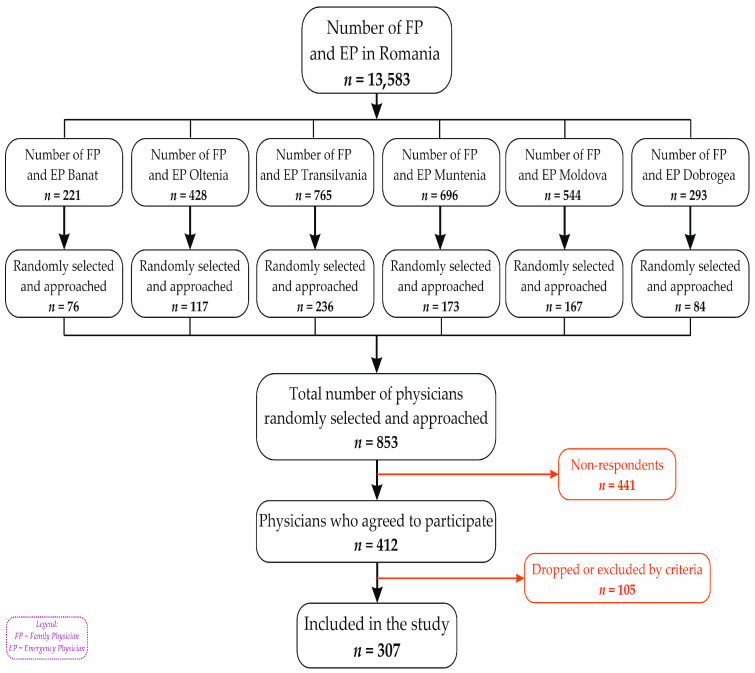
Reporting flow diagram.

To collect a representative national sample of FPs and EPs, a multistage cluster method was used (Figure 1). The territorial division was made in accordance with the geographic regions of Romania (Banat, Dobrogea, Moldova, Muntenia, Oltenia, and Transilvania). During the first stage, one to three counties were selected from each region. County selection was based on the size and development level of the local healthcare infrastructure, ensuring that both well-developed and less-developed counties were represented. In the second stage, the selected counties were divided into urban and rural administrative areas (clusters), and sampling aimed to reflect the actual distribution of physicians across these clusters, ensuring both geographic and specialty representation.

Inclusion criteria comprised being an active physician in one of the two specialties of interest (family medicine or emergency medicine), practicing in Romania, and providing complete responses to all survey items.

Based on population estimates, the required sample sizes were 265 FPs and 217 EPs, assuming a 5% margin of error and a 90% confidence interval [22]. The survey was distributed to approximately 853 physicians through the professional network of the Romanian College of Physicians.

A total of 412 responses were collected, with an overall response rate of 48.3%. Although the response rate was lower than initially expected in some clusters, leading to minor deviations from the planned proportions, the final sample retained a balanced distribution across urban and rural areas. After applying the inclusion criteria, 105 participants were excluded due to incomplete responses (mostly age) or belonging to specialties other than those targeted.

The online questionnaire used in this study comprised 57 items and included both the Maslach Burnout Inventory–Human Services Survey (MBI-HSS) [3] for assessing burnout dimensions, as well as additional sections covering demographic, lifestyle, and occupational variables, as detailed below.

- Demographic data—age, gender, environment (urban/rural).

The ‘environment’ variable was measured through a single self-reported item, where respondents indicated whether they live and practice in an urban or rural area. Classification followed the Romanian National Institute of Statistics definitions [23].

b. Nutritional status, expressed as body mass index (BMI), which was calculated as weight/height^2^ (kg/m^2^), was based on the self-estimations of the participants. Afterwards, the study group was divided based on the BMI value into four categories, in accordance with the World Health Organization’s reference points [24]: underweight (BMI < 18.5); normal weight (BMI between 18.5 and 24.9); overweight (BMI between 25 and 29.9); and obese (BMI > 30, with a further division into 3 stages of obesity: 1st degree 30–34.9; 2nd degree 35–39.9; and 3rd degree > 40).
c. Maslach Burnout Inventory (MBI).

Burnout dimensions were assessed using the Maslach Burnout Inventory–Human Services Survey (MBI-HSS) [3]. This instrument consists of 22 items divided into three subscales: emotional exhaustion (EE, 9 items), depersonalization (DP, 5 items), and personal accomplishment (PA, 8 items). Each item was rated on a 5-point Likert scale ranging from 0 (“never”) to 4 (“every day”). EE scores were obtained by summing the scores of the nine corresponding items, with higher values indicating higher levels of EE. Higher EE and DP scores and lower PA scores reflect higher levels of burnout. No separate stress scale or additional items were used. The term ‘stress’ was used descriptively in the Introduction to refer to general work-related strain, not as a measured variable.

In this study, EE and frustration were treated as distinct constructs: EE was assessed as a core dimension of burnout using the EE subscale, whereas frustration was operationalized through lower scores on the PA subscale, in accordance with previous literature associating reduced PA with higher professional frustration [25].

d. Physical activity (PhyAct).

The survey estimated the level of PhyAct, based on its weekly frequency and its duration. PhyAct was quantified during professional activity: vigorous/moderate PhyAct at work (at least 10 min of intense/moderate effort at once); organized PhyAct (various forms of PhyAct, attended regularly in an organized setting); and unstructured PhyAct (walking, cycling, or unstructured PhyAct around one’s household).

e. Eating behavior. Daily eating habits were documented: number of daily meals; place of eating and type of preferred food/snack; the need to overeat during stressful or tiresome periods; alcohol and energy drink usage depending on one’s degree of exhaustion; and one’s reported importance of meal breaks.

Collected data were initially introduced into a Microsoft Excel database, which was later exported for statistical analysis in IBM SPSS Statistics, Version 20 (IBM Corp., Armonk, NY, USA) and R, Version 4.3.3 (R Foundation for Statistical Computing, Vienna, Austria; https://www.r-project.org). The analysis included descriptive methods, Pearson and Spearman correlations, multiple linear regressions (with adjustment for multicollinearity), alongside comparative tests (Student t, Chi-square, Kruskal–Wallis and ANOVA). The Kolmogorov–Smirnov test was used to assess the normal distribution of quantitative variables (age, weight, height, frequency of weekly PA expressed in minutes/day, number of daily main meals and snacks). Pearson correlation coefficients were applied for variables with an approximately normal distribution, while Spearman coefficients were used for non-normally distributed variables. The strength of correlations was interpreted according to Cohen’s criteria (1988): r = 0.10–0.29 (small), r = 0.30–0.49 (medium), and r ≥ 0.50 (large). The threshold of statistical significance was set at *p* < 0.05.

## 3. Results

The final study cohort consisted of 307 physicians (222 FPs and 85 EPs).

### 3.1. Demographics of the Studies Group

Demographic characteristics of family physicians (FPs) and emergency physicians (EPs) can be found in Table 1. From the gender perspective, a preponderance of women was observed in both categories: 86.9% of FPs and 68.2% of EPs. The gender distribution differs significantly between the two groups. The mean age of the study participants was 53.5 years for FPs and 44.2 years for EPs. The participants lived mostly in urban areas, with a relatively different distribution between groups: 73.9% of FPs; 87.1% of EPs.

### 3.2. Differences Between FP and EP

The comparative analysis of psychosocial indicators (Table 2) reveals significant differences between FPs and EPs. EPs reported higher mean scores for frustration (3.2 vs. 2.1, *p* < 0.001), EE (3.5 vs. 2.3, *p* < 0.001), and interpersonal stress (3.1 vs. 1.8, *p* < 0.001), indicating greater vulnerability within this group. Conversely, FPs reported higher mean values for enthusiasm (2.9 vs. 2.0, *p* = 0.002). Perceptions of EE were also significantly more pronounced among EPs (3.0 vs. 1.7, *p* < 0.001).

After describing the differences between FP and EP, the correlations between burnout dimensions and demographic or behavioral variables were examined.

To identify possible associations between demographic variables and the perceived levels of professional frustration and EE, Spearman correlation coefficients (rho) and statistical significance values (*p*) were calculated (Table 3). Frustration was operationalized through lower scores on the MBI subscale of personal accomplishment (PA), whereas EE represented the standard MBI dimension of emotional exhaustion.

Among FPs, age showed a significant positive correlation with both frustration (ρ = 0.145, *p* = 0.039) and EE (ρ = 0.162, *p* = 0.021), indicating that older physicians tended to report higher levels of professional frustration and EE. Among EPs, correlations between age and frustration (ρ = 0.134, *p* = 0.229) as well as age and EE (ρ = 0.173, *p* = 0.112) were positive but did not reach statistical significance.

For FPs, gender showed no correlation with EE (ρ = −0.115, *p* = 0.044), according to Cohen’s criteria, suggesting that gender differences are negligible. In contrast, no significant correlation was observed between gender and frustration (ρ = −0.073, *p* = 0.255). Regarding EPs, no correlations were observed for either variable—frustration (ρ = −0.049, *p* = 0.657) and EE (ρ = −0.096, *p* = 0.383)—according to Cohen’s criteria.

No significant correlations were identified between the living environment and the frustration or EE scores in either of the two groups, the correlations being similar in direction and amplitude.

BMI did not correlate significantly with any of the analyzed variables. The values were extremely low and insignificant for both FPs and EPs.

### 3.3. Associations of BMI and Living Environment

The distribution of BMI categories, presented in Table 4, varied slightly between the two professional groups (FPs and EPs), as well as between urban and rural living environments. Generally, the most frequent category was overweight, followed by obesity, in both professional groups.

Although BMI distribution varied slightly between urban and rural environments, no significant differences in burnout indicators (frustration and EE) were observed between these groups. However, both variables—living environment and BMI—are potentially relevant to burnout. Urban environments are typically associated with higher workloads and systemic pressures, which may increase burnout risk, whereas rural environments can generate frustration due to limited access to diagnostic resources. Similarly, overweight and obesity may be associated with lower physical endurance and higher fatigue, potentially amplifying burnout symptoms. These considerations justify their inclusion in the analysis, even if no significant effects were observed in this cohort.

Among EPs, the proportion of normal weight participants was relatively similar between rural (27.3%) and urban (25.7%) living environments. Obesity was more common in urban areas (35.1%) than in rural areas (27.3%), while more underweight people, though few, were present in rural areas (9.1%).

For FPs, overweight and obese participants dominated both environments: 77.6% of rural inhabitants and 65.8% of the urban ones. The number of underweight participants is practically zero in rural areas (0%) and very low in urban areas (2.4%).

There was no clearly differentiated distribution according to living environment, but rather a slight trend towards a higher proportion of normal weight participants in urban areas for FPs and participants with obesity in urban areas for EPs. At the same time, cases of underweight were marginal, indicating an overall nutritional status above normal in both occupational groups.

### 3.4. Physical Activity and the Perceived Level of Frustration and EE

In terms of PhyAct patterns, EPs reported slightly higher engagement in vigorous exercise and recreational sports compared to FPs, while FPs more frequently incorporated PhyAct into their daily routines (e.g., walking, gardening). Overall, both groups reported low-to-moderate levels of structured PhyAct, with no significant differences between groups.

Physical activity was assessed using a 5-point Likert scale (0 = never, 4 = always) covering different types of activities, such as structured exercise, cardiovascular workouts, daily physical routines, and recreational sports. The resulting scores reflect frequency rather than duration. Median scores (min–max) for physical activity items among family physicians were between 0.00 (0–4) and 2.00 (0–4), while among emergency physicians, they ranged from 0.00 (0–4) to 3.00 (0–4). Emergency physicians reported slightly higher scores for vigorous and recreational activities, whereas family physicians more frequently reported incorporating physical activity into their daily routines (e.g., walking, gardening). Statistically significant differences between groups were observed for cardiovascular exercise (*p* = 0.024) and strength/resistance training (*p* < 0.001), with emergency physicians engaging more frequently in these types of activities.

The analysis of the correlation between various forms of PA and the perceived levels of frustration and EE among respondents highlighted several statistically relevant aspects, as presented in Table 5.

Strength/endurance exercises and participation in recreational physical hobbies (hiking, dancing, etc.) showed moderate correlations with professional frustration (ρ = 0.379 and ρ = 0.343, respectively), while general involvement in PhyAct, cardiovascular exercise and incorporating physical exercise into the daily routine (such as walking or gardening) recorded modest correlations.

EE correlated negatively with PhyAct, suggesting that an active lifestyle has a modest protective effect against mental exhaustion. However, no correlations were observed (ρ between −0.112 and −0.122), according to Cohen’s criteria, indicating that the associations are negligible in magnitude. Engaging in recreational physical hobbies also had a close negative correlation of significance (ρ = −0.110, *p* = 0.054).

### 3.5. Eating Behavior

Following the link between fast food consumption and the level of professional frustration, a statistically significant difference in the level of frustration was identified between participants who regularly consumed fast food and those who did not (*p* = 0.04397).

In the multiple linear regression model, the combined consumption of dairy and biscuits had a positive coefficient of +3.22, with a *p*-value = 0.084. Although this result does not reach the threshold of statistical significance, it does indicate a possibly relevant trend that deserves further investigation.

Respondents who stated that the last meal of the day was the “afternoon snack” had a negative coefficient of −1.42, with a *p* = 0.081, indicating a trend of inverse association between this eating behavior and frustration, as presented in Table 6.

### 3.6. Significant Correlations with Work-Related Frustration

To investigate the relationship between perceptions related to professional activity and the level of exhaustion reported by respondents, Spearman correlations between several psychosocial items and the overall frustration score were calculated, according to Table 7.

Items positively correlated with frustration included indicators of mental and physical overload. The strongest associations were found for “Working with people all day is really a strain for me” (ρ = 0.735, *p* < 0.001) and “I feel like I’m at the end of my rope” (ρ = 0.696, *p* < 0.001), suggesting that excessive relational strain and extreme fatigue are closely linked to frustration. Perceived workload (“I feel I’m working too hard on my job”, ρ = 0.644, *p* < 0.001) and daily intense human interactions (“Working with people directly puts too much stress on me”, ρ = 0.624, *p* < 0.001) also showed significant positive correlations. Overall, high positive coefficients (ρ > 0.6) underscore the strong link between perceived overload and professional frustration.

Subgroup analyses were also performed to examine potential differences between FP and EP. The overall pattern of associations was similar in both groups, with no statistically significant differences between correlation coefficients.

Conversely, several items showed negative correlations with frustration, suggesting potential protective factors. General vitality (“I feel very energetic”, ρ = −0.383, *p* < 0.01), the ability to create a relaxed atmosphere with patients (“I can easily create a relaxed atmosphere with my recipients”, ρ = −0.354, *p* < 0.01), and enthusiasm derived from interpersonal relationships (“I feel exhilarated after working closely with my recipients”, ρ = −0.192, *p* < 0.01) were all inversely associated with frustration, indicating that positive energy and relational competence may mitigate its occurrence.

No significant differences were observed between FPs and EPs regarding the strength of these correlations.

All variables included in the analysis were derived from Likert-type questionnaire items (0–4), treated as ordinal variables. Therefore, Spearman correlation coefficients were used.

**Figure 2:**
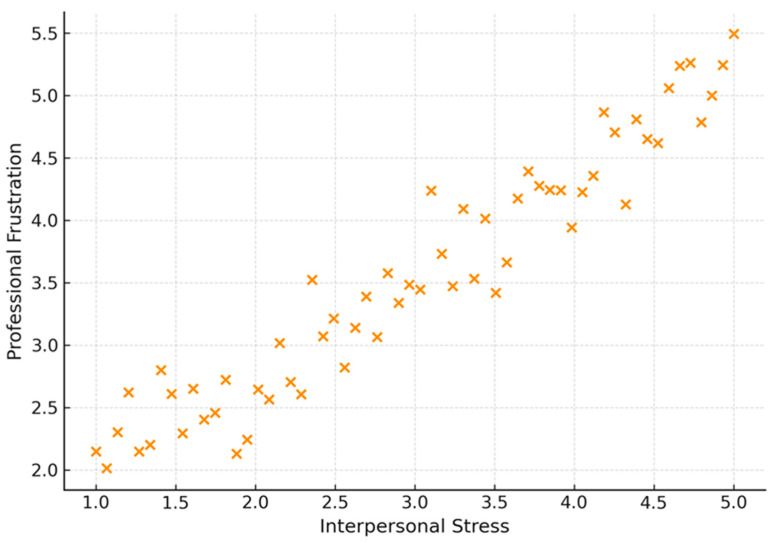
Correlation between interpersonal stress and professional frustration.

A significant positive correlation was highlighted between the stress caused by interpersonal interaction and professional frustration (ρ = 0.570, *p*-value < 0.001). This highlights the role and influence of strained relationships on physicians’ emotional discomfort. The more stressful a physician perceived social interactions to be, the higher their frustration score, as shown in Figure 2.

### 3.7. Regression Model for the Level of Professional Frustration

To explain the variation in professional frustration scores according to selected psychosocial predictors, a linear regression model was performed (Table 8). Enthusiasm emerged as a significant protective factor (B = −2.69, *p* = 0.0347; OR = 0.068, 95% CI: 0.025–0.181), whereas interpersonal stress was a strong positive predictor (B = 1.01, *p* = 0.0166; OR = 2.746, 95% CI: 1.030–7.316). Treating patients in an impersonal or objectified manner was also associated with increased frustration (B = 0.61, *p* = 0.0408; OR = 1.840, 95% CI: 0.691–4.904), though with a lower predictive strength.

### 3.8. Regression Model for Exhaustion

To identify relevant predictors of emotional exhaustion, a linear regression model was performed, including perceived daily fatigue, frequent frustration, and perceptions of extreme exhaustion as independent variables (Table 9). Frequent perceptions of being at the limit of one’s resources constituted the most powerful predictor (B = 1.26, *p* < 0.001; OR = 3.524, 95% CI: 1.322–9.395). Daily evening fatigue also showed a significant positive association (B = 1.20, *p* = 0.0112; OR = 3.321, 95% CI: 1.313–8.392). Furthermore, frequent professional frustration contributed significantly to higher exhaustion scores (B = 0.73, *p* = 0.0003; OR = 2.075, 95% CI: 1.383–3.111), illustrating the strong interdependence between these constructs.

## 4. Discussion

The aim of this study was to assess burnout among family physicians (FPs) and emergency physicians (EPs) in Romania, and to examine how demographic and occupational factors contribute to their resilience. The prevalence of burnout identified is broadly consistent with findings from other European countries, confirming that physician distress is not confined to one region but reflects a continental trend. This reinforces the idea that burnout should be considered a European public health concern rather than a purely local problem, especially within healthcare systems facing workforce shortages and sustained psychosocial stressors [26]. This perspective aligns with recent calls for coordinated EU-level strategies to address healthcare workforce well-being and retention. At the same time, the Romanian context shows distinctive features. The high proportion of female primary care physicians and the relatively senior age composition of FPs may amplify vulnerability to exhaustion. These aspects highlight the need for tailored interventions that address both demographic and professional realities.

Our results are consistent with the findings of the WHO MeND 2025 report, which documented significant associations between unsafe working conditions and mental health problems among physicians and nurses across Europe. The report underlines that long working hours, insufficient resources, and institutional stress contribute to psychological symptoms and decreased well-being among healthcare workers. These findings support and contextualize the relationships identified in our study between frustration, emotional exhaustion, and psychosocial factors, offering a valuable European comparative perspective [21].

While previous research has examined EP in local settings, our work is the first to include both professional groups in a national survey, thus offering broader comparability and stronger epidemiological relevance regarding primary care providers. The findings are applicable not only locally but also for other European health systems dealing with workforce shortages and organizational strain.

From a wider European perspective, the results underscore the connection between burnout, physician migration, patient safety, and the long-term resilience of healthcare systems. Tackling burnout requires coordinated strategies that extend beyond national borders and align with European health policy priorities.

The results of the current study provide a detailed image of the stress levels of primary care providers in Romania, identifying dissimilarities between family and emergency physicians in terms of professional frustration, emotional exhaustion, interpersonal stress and their psychological resources. These findings are in accordance not only with the structural characteristics of the Romanian healthcare system—marked by high workloads, limited resources, and urban–rural disparities—but also with international literature on burnout among healthcare professionals [1,27]. A study conducted by the Bucharest College of Physicians [28] concluded that 36% of physicians were at increased risk of developing burnout syndrome, while 55% and 52% of the total sample presented personal and professional burnout, respectively—percentages higher than those reported in France (49%) or Germany (35–38% for FP and up to 40% for emergency medical personnel) [29,30]. Comparable levels have also been reported among emergency physicians in Ukraine, highlighting similar challenges faced in Eastern European healthcare systems [20].

Another study focusing on resident physicians [31] showed that burnout was associated with absenteeism, low job satisfaction, and diagnostic errors. Although these factors were not measured in our study, the burnout patterns we identified—including strained patient relationships and reduced professional enthusiasm—are consistent with previous findings, suggesting that similar consequences may occur. The results of the present study extend these observations, highlighting the specific role of strained relationships with patients and decreased professional enthusiasm.

In this study, “Frustration” was operationalized through lower scores on the personal accomplishment (PA) subscale of the Maslach Burnout Inventory, in line with previous literature linking reduced PA to increased professional frustration and unmet goals [27,32]. This reflects physicians’ perceived mismatch between effort and outcomes. The strong correlations found between frustration (as derived from PA) and EE support the notion that frustration may function as both a precursor and an amplifier of EE, especially in high-demand clinical settings.

Burnout syndrome among emergency medicine professionals constitutes a notable challenge, with its levels being high both pre- and post-pandemic. In a pre-pandemic literature review, the share of affected personnel ranged from 25% to 77% [33], while an international post-pandemic study reported a 62% impairment rate [34].

Regarding the demographic characteristics of the present cohort, these are in agreement with the tendencies presented in the literature. The gender distribution differs significantly between the two groups (FP and EP), with the dominance of female physicians reflecting the current demographic trends in the Romanian medical system for both specialties. A similar gender proportion was observed in other Romanian studies [11,12,13].

Despite this dominance, our research did not identify gender as a relevant differentiating factor for burnout levels (Frustration and EE), even though previous studies have reported emotional coping variations [35,36] and higher EE among non-male physicians during the COVID-19 pandemic [37].

Analyzing the age distribution of the two professional groups, a seniority of the FPs was noted, yet it should be considered that the emergency medicine specialty is a relatively young one, both nationally and internationally [38,39], and the first generations of EPs are still active HCPs. Correlating age with respondents’ level of EE and frustration, a significant result was determined for FPs. Though modest, this result suggests that older physicians feel professional stress slightly more intensely, probably due to the accumulation of chronic factors (bureaucracy, professional burnout, sustained physical effort) [40,41]. The lack of correlation for EPs is in line with the literature, where it was found that older age is a protective factor for burnout, especially in the context of the COVID-19 pandemic, when young professionals contributed more to the management of the pandemic and suffered from lower levels of social support [34,37].

The predominantly urban location of EPs can be explained by the concentration of emergency medical services in urban centers, while noting that the present study did not follow the activity of those EPs who exclusively serve the pre-hospital sector, which also covers rural areas. However, the living environment did not directly influence the perceived psychological stress, possibly due to the balancing of other factors (e.g., increased workload in urban areas, professional isolation in rural ones).

These findings are consistent with the existing literature, which indicates that urban settings are often associated with higher workloads and organizational pressures leading to burnout, whereas rural environments may generate frustration due to infrastructural and resource limitations [42,43].

All types of physical activity correlated positively and significantly with frustration (ρ between 0.164 and 0.379), suggesting that as the perceived level of frustration increases, those individuals have an increased tendency to turn to PhyAct—either as a coping mechanism or as a reflex component of an active lifestyle—despite psychological discomfort. Similar results were reported by De Oliveira Jr. et al. (2019) [44], who highlighted the beneficial role of exercise in regulating emotions in HCPs.

PhyAct seems to be associated with lower levels of mental exhaustion, even if the effect is weak. These findings are congruent with those from the study by Montgomery et al. (2021) [45], which suggest a protective role of physical movement for the psychological well-being of physicians. This relationship may reflect both a strategy of emotional regulation through physical exertion and the need for physiological discharge associated with states of psychological tension.

The results suggest that PhyAct has a complex relationship with burnout: frustrated people tend to exercise more often, while EE seems to be lower in physically active people. This dual role—as a compensatory mechanism and a protective factor—of PhyAct is worth exploring in future interventions to support mental health among HCPs.

According to our results, it can be assumed that having a regular meal schedule could be associated with less frustration, yet this association was not statistically significant, only suggestive. In terms of eating behaviors, the data implies that the habit of frequently consuming fast food is associated with increased levels of frustration. This association may reflect irregular daily routines and an unhealthy coping mechanism, in line with the observations of Utter J. (2023) [46] on the connection between unbalanced eating and occupational stress. There could be an association between the preference for dairy and biscuit snacks and a higher level of frustration, possibly as a poor emotional regulation mechanism.

Direct patient care and increased workload were significantly correlated with EE, which supports the literature on burnout in HCP [27].

As for professional frustration, we observed that it is fueled by the stress caused by daily interpersonal stress and perceived increased workload.

Our data suggest that EE is strongly linked to the perception of increased professional workload and interpersonal stress. The perceptions of good vitality, good relationship skills, and increased professional enthusiasm act as positive, protective factors for the respondents. This observation is also supported by West et al. (2022) and Shanafelt et al. (2015) [1,5], whose studies reinforce the idea that increased workload and relational pressure magnify burnout, whilst vitality, enthusiasm, and positive interactions/relationships act as protective factors.

The correlation coefficients, all statistically significant, validate these interactions and may guide interventions aimed at reducing EE in professions intensely exposed to direct patient care. According to a pre-pandemic study [47], emergency HCPs (physicians and nurses) reported a higher perception of social stress and emotional dissonance compared to acute care surgeons, with the impairment being reported as stronger by EPs when compared to nurses. Additionally, over 50% of EPs and emergency nurses reported “having to suppress their feelings ‘almost every day’ to ‘several times a day’”.

Several psychological scores were significantly higher in EPs (frustration, EE, interpersonal stress, and feeling of extreme exhaustion). These results confirm the increased vulnerability of emergency HCPs, who are subject to intense demands, unpredictable work rhythms, and lack of resources, as supported by various research [2,35]. These data encourage the hypothesis that the particularities of each specialty directly influence the psychological state of HCPs.

The multiple regression model showed that work-related enthusiasm was a significant negative predictor, meaning that an increased level of enthusiasm is associated with a significant decrease in frustration.

The increase in stress due to direct human interactions (patients, colleagues, or others) was associated with a significant expansion of the frustration score, indicating that social stress is an important risk factor.

Perceiving patients in dehumanized terms was associated with an increased level of frustration, thus confirming the observations of Dyrbye et al. (2014) on the role of strained relationships in the onset of burnout [32]. This behavior may reflect a defense mechanism in conditions of increased workload, yet it negatively impacts physicians’ emotional well-being.

Professional frustration was influenced by interpersonal enthusiasm (MBI item “I feel exhilarated after working closely with my recipients”) as a protective factor, interpersonal stress as a risk factor, and tendencies of patient dehumanization. All the included predictors were statistically significant (*p* < 0.05), confirming the robustness of the results. These findings support the idea that professional exhaustion is the consequence of the accumulation of negative emotional factors and that the lack of positive resources, such as enthusiasm, amplifies the risk of burnout. This mechanism has also been described by Maslach & Leiter (2016), who emphasized the importance of enthusiasm and psychological resources for burnout prevention, but also by Shanafelt et al. (2015), who showed that relational pressure and loss of professional meaning significantly increase physicians’ vulnerability [5,27].

The results indicate a clear overlap between frustration and EE, especially for EPs. The strongest common predictors were interpersonal stress, extreme fatigue, and lack of enthusiasm. Several national studies also confirm these trends. According to the Romanian College of Physicians, a 2023 report stated that 36% of physicians are at high risk of burnout, with EE as the predominant component (58%) [28]. Dimitriu et al. (2020) reported an alarming degree of burnout (≈76%) among resident physicians, especially in non-COVID wards [48]. Moșcu et al. (2022) highlighted that more than one third of the nurses in a level-3 emergency department experienced severe burnout and showed a direct relationship between EE and job satisfaction [49]. The study conducted by Puia A. et al. (2021) [50] on a cohort of resident and specialist physicians identified increased levels of EE and a high prevalence of depersonalization symptoms. Similar trends were emphasized by other authors (Corlade et al., 2022; Olariu et al., 2021), in both EPs and FPs, especially in the absence of effective organizational support [12,51].

## 5. Limitations

The limitations of our study should be taken into account when interpreting the results. First, the cross-sectional design did not allow for establishing causal relationships between variables. Although we have identified significant associations between psychosocial factors and levels of frustration or EE, we could not determine the direction of these relationships. This aspect is recognized in the methodological literature, so cross-sectional studies cannot test causal hypotheses without subsequent longitudinal investigations [52,53].

Self-reporting data collection can generate biases, such as memory errors or the effect of social desirability bias, in which respondents tend to provide answers perceived as socially acceptable [54]. This phenomenon is frequently encountered in studies on mental health and personal behaviors, where responses are subject to participants’ sensitivity.

Another limitation is the exclusively online distribution of the survey. This method can generate uneven sampling, with an over-representation of digitally engaged physicians and an under-representation of those from rural areas or with limited digital access [55].

Additionally, the lack of objective indicators (such as electronic monitoring of physical activity, physiological measures of stress or direct observations of occupational behaviors) reduced estimation accuracy and limited data triangulation [54,56]. In the future, studies should integrate both objective quantitative methods and qualitative approaches for a more complete understanding of the phenomenon.

Although all three MBI subscales were collected, this study focused on emotional exhaustion and professional frustration, as these were most relevant to the research objectives and local context. Detailed analyses of depersonalization and personal accomplishment were beyond the scope of this manuscript and will be addressed in future work.

A limitation of this study is the absence of a validated frustration-specific scale (e.g., Basic Psychological Need Frustration and Satisfaction Scale (BPNFSF)), which could complement the frustration score derived from MBI subscales in future research.

Forthcoming studies could benefit from a longitudinal approach, with larger and better distributed samples, the integration of objective measurements, and the design and assessment of organizational interventions aimed at reducing burnout.

## 6. Conclusions

Burnout among primary care physicians should be recognized as both a national challenge for Romania and a European public health issue. Effective strategies to support workforce resilience are essential, in line with the EU’s agenda on health workforce sustainability and the Sustainable Development Goal 3 (Good Health and Well-being) [57,58,59].

The study highlights an increased incidence of emotional exhaustion and professional frustration among primary care providers, more pronounced in emergency physicians than family physicians. The main predictors of frustration were interpersonal stress, workload, and the tendency to treat patients in a dehumanized way, while professional enthusiasm and relational skills proved protective factors. Physical activity was associated with both a slight reduction in emotional exhaustion and an increase in frustration, and unhealthy eating habits correlated with higher levels of frustration.

These results emphasize the need for differentiated interventions for each specialty, with a focus on reducing interpersonal stress, managing workload, and promoting positive resources. The implementation of organizational burnout prevention programs, adapted to the particularities of each specialty, as well as encouraging professionals to adopt individual strategies to promote wellness, can significantly contribute to improving the well-being of doctors and, consequently, increase the quality of care.

## Figures and Tables

**Table 1:** Demographic characteristics of family physicians (FPs) and emergency physicians (EPs).

	Family Physicians		Emergency Physicians	
Participants, n	222		85	
Gender, %	F	M	F	M
	86.9	13.1	68.2	31.8
Age, Mean ± SD, years	53.5 ± 10.0		44.2 ± 10.5	
Living environment, %	Urban	Rural	Urban	Rural
	73.9	26.1	87.1	12.9

**Table 2:** Comparison of psychosocial scores between family physicians and emergency physicians.

Variable	FP	EP	*p*-Value
I feel frustrated by my job	2.00 (0–4)	2.00 (0–4)	<0.001
I feel burned out from my work	2.00 (0–4)	2.00 (0–4)	<0.001
Working with people directly puts too much stress on me	2.00 (0–4)	2.00 (0–4)	0.024
I feel exhilarated after working closely with my recipients	2.00 (0–4)	2.00 (0–4)	0.002
I feel like I’m at the end of my rope	2.00 (0–4)	2.00 (0–4)	<0.001

FP = family physician; EP = emergency physician; values are presented as medians (min–max); *p* < 0.05 was considered statistically significant. Although median values were similar across groups, Mann–Whitney U tests indicated significant differences in the distributions of responses for several items.

**Table 3:** Correlations between demographic variables and levels of frustration and emotional exhaustion.

Factor	Family Physician				Emergency Physician			
	Frustration		EE		Frustration		EE	
	ρ	*p*	ρ	*p*	ρ	*p*	ρ	*p*
Age	0.145	0.039	0.162	0.021	0.134	0.229	0.173	0.112
Gender	−0.073	0.255	−0.115	0.044	−0.049	0.657	−0.096	0.383
Environment	−0.057	0.381	−0.045	0.493	−0.098	0.373	−0.134	0.214
BMI	−0.012	0.855	−0.003	0.964	−0.019	0.869	−0.008	0.942

EE = emotional exhaustion; BMI = body mass index; ρ = Spearman correlation coefficient; *p* < 0.05 was considered statistically significant.

**Table 4:** Distribution of BMI categories by living environment and professional group.

BMI Category	Urban FP	Rural FP	Urban EP	Rural EP
Normal, %	31.7	22.4	25.7	27.3
Obese, %	34.2	37.9	35.1	27.3
Underweight, %	2.4	0.0	4.1	9.1
Overweight, %	31.7	39.7	35.1	36.3

BMI = body mass index; FP = family physician; EP = emergency physician.

**Table 5:** Associations between types of physical activity, frustration, and emotional exhaustion.

Type of Physical Activity	Frustration		Emotional Exhaustion	
	*ρ*	*p*-Value	*ρ*	*p*-Value
I engage in physical activities, such as going to the gym or attending fitness classes	0.186	0.001	−0.122	0.032
I participate in cardiovascular exercises like jogging, swimming, or cycling	0.273	<0.001	−0.112	0.050
I engage in strength training or resistance exercises, such as lifting weights	0.379	<0.001	−0.168	0.005
I incorporate physical activity into my daily routine, like walking or gardening	0.164	0.004	−0.107	0.045
I participate in recreational sports or physical hobbies like hiking, dancing	0.343	<0.001	−0.154	0.009

*ρ* = Spearman correlation coefficient; *p* < 0.05 was considered statistically significant.

**Table 6:** Associations between eating behaviors and professional frustration.

Food Variable	r	*p*-Value
Snack type = Dairy/Biscuits	+3.22	0.084
Last meal = afternoon snack	−1.42	0.081

r = Pearson correlation coefficient; *p* < 0.05 was considered statistically significant.

**Table 7:** Correlations between professional activity items and frustration scores.

Variable	ρ	*p*-Value
I feel like I’m at the end of my rope	0.696	<0.001
Working with people all day is really a strain for me	0.735	<0.001
I feel I’m working too hard on my job	0.644	<0.001
Working with people directly puts too much stress on me	0.624	<0.001
I feel very energetic	−0.383	<0.01
I feel exhilarated after working closely with my recipients	−0.192	<0.01
I can easily create a relaxed atmosphere with my recipients	−0.354	<0.01

ρ = Spearman correlation coefficient; *p* < 0.05 was considered statistically significant.

**Table 8:** Regression model of psychosocial predictors of professional frustration.

Predictor	B	*p*	OR	CI Min	CI Max
(Intercept)	4.90	0.0187	134.290	50.400	357.809
Enthusiasm (maximum level)	−2.69	0.0347	0.068	0.025	0.181
Interpersonal stress (maximum level)	1.01	0.0166	2.746	1.030	7.316
Treating patients as “objects”	0.61	0.0408	1.840	0.691	4.904

*p* < 0.05 was considered statistically significant; CI—confidence interval; OR—odds ratio; B—non-standardized regression coefficient.

**Table 9:** Regression model of psychosocial predictors of emotional exhaustion.

Predictor	B	*p*	OR	CI Min	CI Max
Feeling at the end of one’s rope (frequently)	1.26	<0.001	3.524	1.322	9.395
Exhaustion in the evening (daily)	1.20	0.0112	3.321	1.313	8.392
Frustration (frequently)	0.73	0.0003	2.075	1.383	3.111

*p* < 0.05 was considered statistically significant; CI—confidence interval; OR—odds ratio; B—non-standardized regression coefficient.

## Data Availability

The data are not publicly available due to privacy and ethical restrictions. Aggregated results are available from the corresponding author upon reasonable request.

## Abbreviations

The following abbreviations are used in this manuscript:

BMI	Body mass index
CI	Confidence interval
DP	Depersonalization
EE	Emotional exhaustion
EP	Emergency physician
FP	Family physician
HCP	Healthcare professionals
MBI	Maslach Burnout Inventory
OR	Odds ratio
PA	Personal Accomplishment
PhyAct	Physical activity
